# Gender in Science and Engineering Faculties: Demographic Inertia Revisited

**DOI:** 10.1371/journal.pone.0139767

**Published:** 2015-10-21

**Authors:** Nicole R. Thomas, Daniel J. Poole, Joan M. Herbers

**Affiliations:** 1 Comprehensive Equity at Ohio State, The Ohio State University, Columbus Ohio, United States of America; 2 Mathematics Department, The Ohio State University, Columbus Ohio, United States of America; Northwestern University, UNITED STATES

## Abstract

The under-representation of women on faculties of science and engineering is ascribed in part to demographic inertia, which is the lag between retirement of current faculty and future hires. The assumption of demographic inertia implies that, given enough time, gender parity will be achieved. We examine that assumption via a semi-Markov model to predict the future faculty, with simulations that predict the convergence demographic state. Our model shows that existing practices that produce gender gaps in recruitment, retention, and career progression preclude eventual gender parity. Further, we examine sensitivity of the convergence state to current gender gaps to show that all sources of disparity across the entire faculty career must be erased to produce parity: we cannot blame demographic inertia.

## Introduction

Women have historically been under-represented among science and engineering faculty [1, 2]; even today, disciplines that have produced PhDs at parity for decades have fewer women than men faculty [3, 4]. Furthermore, women become increasingly under-represented in higher-ranking positions, including Professors, Department Chairs, and Deans [1, 5–9]. What are the causes of these gender gaps and how can we work towards parity? Numerous factors produce the gaps, including family constraints, institutional rigidity, and systemic unconscious bias [2, 4–6, 8, 10–14]. We can examine those factors at important transition points, from first hire into a tenure-track position through tenure and promotion to Associate Professor and then on to Professor.

Recruitment of women to tenure-track positions is most sensitive to the quantity and quality of their applications for such positions as well as the behavior of selection committees. Women are under-represented in the application pool, relative to the proportion of PhD earners in science and engineering, which shows the postdoctoral years are critical to women’s persistence [1, 4, 6, 7, 11]. Search and selection processes do not appear to disadvantage women, so increasing their representation in the pool is the primary imperative for effective recruitment [4, 15, 16].

One large-scale analysis has shown that many men and women leave their faculty positions (and sometimes the discipline) within 10 years of hire [17]. Recruitment costs, especially in the STEM disciplines (science, technology, engineering, and mathematics), have escalated and competition for faculty has intensified in an era of dwindling support; when a recent recruit resigns, those startup costs cannot be fully recouped. These realities, in turn, have prompted research institutions to focus on the fiscal outcomes of faculty attrition [17]. Studies of faculty satisfaction and intent to leave have shown that local department culture and institutional work-life policies are key to faculty retention [18]. Retention has emerged as an institutional priority over the last decade, yet surprisingly little attention has been paid to actual faculty demographics across entire careers.

A corollary of retention is continued professional development. Because achieving tenure and promotion to Associate Professor is the most critical transition in a faculty member’s career, considerable research has focused on the pre-tenure years [1, 11, 18]. By contrast, the continued development of Associate Professors through the remaining 30+ years of the career has been neglected. Understanding of how faculty move through the post-tenure years to achieve promotion to Professor is thin, with few guideposts to help faculty or their administrators [19–23].

We do know that, once women are hired into tenure-track positions, their careers develop differently (14). More specifically, careers of STEM faculty show starkly gendered patterns. Not only are women under-represented on STEM faculties, especially in research-intensive universities, but their job satisfaction is lower [10, 18, 24]. Movement towards the tenure decision shows gender gaps as well [1, 6, 11, 14]; studies of retention show conflicting patterns [4, 17], as does the pathway to Professor [1, 25].

Given the preponderance of men on faculties today, even aggressive hiring and promotion might take a generation to produce gender parity: this lag between gender-neutral hiring and full representation is termed demographic inertia [5, 9, 26–28]. Demographic inertia predicts slow change in faculty composition, and previous studies have focused on the timeframe for and approach to parity (9,16,25–27). Yet studies of demographic inertia beg the question whether current practices can ever produce gender parity. By using a semi-Markov approach, we show that current patterns of recruitment, retention and career progression will never accomplish that goal: demographic inertia cannot forever take the blame for women’s under-representation on STEM faculties.

## Materials and Methods

**Fig 1:**
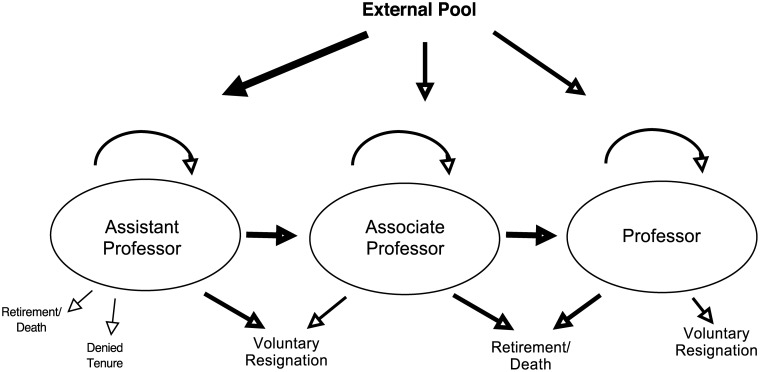
Model of faculty flux. Our analysis of faculty flux examined transitions between possible states. Arrow size denotes observed relative magnitude of different transitions across a seven-year interval.

We model faculty recruitment, retention, and promotion as a series of transitions (Fig 1). Those transitions fit a semi-Markov process, which itself can converge to a steady state. For our study we used demographic data for tenure-track faculty in the natural sciences and engineering at a large research university in the US to examine transitions among states (Fig 1). The resulting transition matrices, further described below, were used to calculate the proportions of faculty at convergence.

Rosters of tenure track and tenured faculty in engineering and natural sciences were provided by the institution for 5 time points (1998, 2002, 2005, 2009, 2012). These snapshots allowed us to define three cohorts over seven-year intervals (1998–2005, 2002–2009, 2005–2012) *(28)*. We tracked each individual from start to end of a 7-year period, and assigned each to a category at the end of that period: Assistant Professor, Associate Professor, Professor, resigned, denied tenure, retired/died. These 6 categories and their frequencies were then used to construct a transition matrix that showed proportions of individuals from each category at time *t* who ended up in the final categories at *t+7* years, with men and women considered separately (see Table 1). These data allowed us to examine retention and promotion across the three faculty cohorts.

**Table 1:** Numbers of individual faculty used to compute transition matrix data for the three cohorts.

Initial Status	Status 7 years later	1998–2005		2002–2009		2005–2012	
		Women	Men	Women	Men	Women	Men
Assistant Professor	Prom to Assoc	6	43	13	45	12	36
	Prom to Full	2	4	4	5	5	15
	Denied tenure	0	3	0	6	0	4
	Resigned	6	14	6	9	10	9
	Retired/Died	1	0	0	0	0	0
	Still in Rank	1	1	1	2	1	1
Associate Professor	Prom to Full	4	59	9	68	10	48
	Still in Rank	5	71	13	77	12	51
	Resigned	2	15	3	4	2	7
	Retired/Died	0	14	2	25	1	25
Full Professor	Still in Rank	9	212	13	210	15	188
	Resigned	0	18	0	21	2	13
	Retired/Died	1	58	6	91	4	102

Impossible transitions (such as full Professor to Assistant Professor) are not listed.

We then modeled these transitions via a semi-Markov process. Our fundamental equation tracks faculty demographics across the seven-year period as follows:

Ω(t+7)=ΑΩ(t)+Β

where Ω(*t*) is a column vector with six elements *ω*_*i*_(*t*) that denote the numbers of Assistant Professors, Associate Professors, Professors, those denied tenured, those who voluntarily resigned, and those who retired/died. The 6x6 transition matrix A is populated with proportions obtained from data as in Table 1. This matrix has a number of zeroes for impossible transitions such as Professor to Assistant Professor. Finally, B is a column vector denoting the numbers hired into the three ranks, with zeroes for the last three elements that denote tenure denials, resignations, and retirements/death.

If we assume that the transition matrix A and recruitment vector B remain constant, then we can iterate the equation across time to project faculty composition (see Supplemental Information for a full explanation of methodological details). As iteration proceeds, the projections of faculty demographics in future time periods become more and more similar. Indeed, given sufficient time, this semi-Markov process drives the numbers of faculty by rank and gender to converge to a steady state:

$$\underset{t\to \infty }{\text{lim}}\tilde{\Omega }(t)={\left(Ι-\tilde{Α}\right)}^{-1}\tilde{Β}=\tilde{\Omega }$$

This convergence property allows us to predict the ultimate proportions of men and women faculty of each rank ($\tilde{\Omega }$), which is a function of the modified recruitment vector $\tilde{Β}$ and the career transition matrix $\tilde{Α}$. A fuller explanation is given in the S1 Text.

The Ohio State Human Subjects Institutional Review Board approved this study. Because we used institutional data, informed consent was not required.

## Results

### Faculty Flux

**Fig 2:**
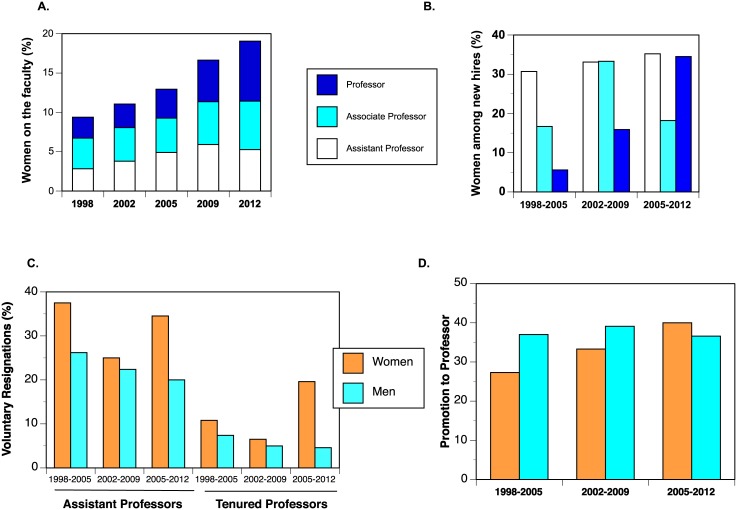
Faculty flux across the three cohorts. A. Gender representation of the faculty across time. B. Recruitment of women to the faculty C. Voluntary resignations among faculty. D. Promotion rates for Associate Professors

Across the entire timeframe of analysis (1998–2012), women’s presence on the STEM faculty steadily grew (Fig 2A). Most important, women’s representation among the highest rank of Professor tripled over this time period. Indeed, women were recruited to the faculty at rates exceeding their current representation, showing that recruitment at all ranks drove the overall increase (Fig 2B). Yet we simultaneously uncovered gender gaps in persistence and career progression. Women faculty, both pre- and post-tenure, resigned voluntarily at higher rates than men in each of the three cohorts (Fig 2C); a retention gender gap persisted over these 15 years. The vast majority of Assistant Professors who were considered for promotion with tenure received positive results; indeed, no woman was denied tenure in the entire dataset (Table 1). The retention gender gap therefore resulted from more voluntary departures of women than men: relatively poor retention of female Assistant Professors counteracted enhanced recruitment. Retention at higher ranks (Fig 2C) was measured as a function of voluntary resignations of tenured Associate Professors and Professors (ignoring retirements and deaths). A gender gap again characterized each cohort, and was largest in the third.

Once tenured, female Associate Professors were promoted at lower rates than men in the first two cohorts; this promotion gender gap was erased in the third cohort (Fig 2D). Even so, only 40% of Associate Professors of either gender had been promoted to Professor within any seven-year period, which suggests that systemic issues impede career progression for both men and women at this institution.

**Fig 3:**
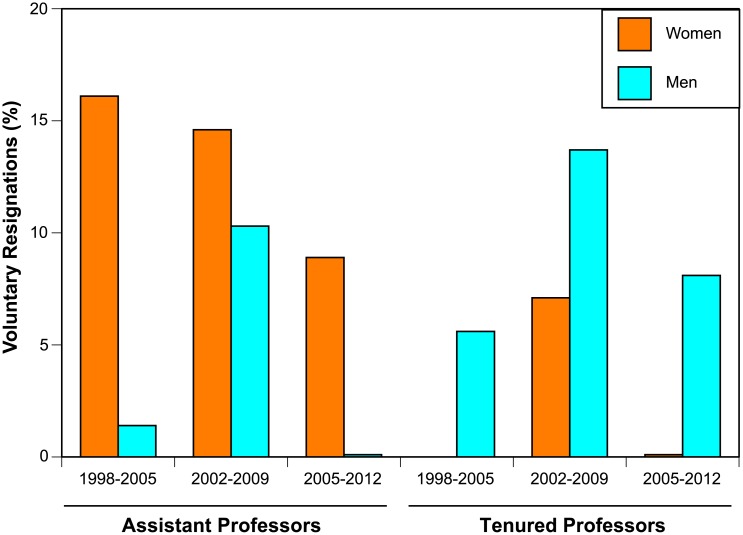
Resignations for faculty hired between census points. Resignations within the same time period (i.e. within 1–6 years of hire).

Finally, we further examined values for faculty recruitment, i.e. values for the vector B above. The analysis above failed to capture the small but informative number of faculty who were hired after the start of a period (at time *t*) and who left before the end of that period (at time *t+7*). These interim resignations (Fig 3) were aggregated across all these individuals, even though their time on the faculty varied from 1 to 6 years. Despite vigorous hiring of women Assistant Professors (Fig 1B), their short-term retention lagged behind that of men: up to 15% of the new female Assistant Professors resigned between census periods, i.e. within 1–6 years of hire (Fig 3). Associate and full Professors represented about 20% of all the interim hires; for this small number, senior women had higher retention rates than did senior men (Fig 3). Faculty who were hired and gone between census points represent substantial retention losses for the institution.

### Simulations

Together, the gender gaps in recruitment, retention, and promotion suggested that demographic inertia was not solely responsible for the under-representation of women on the STEM faculty. Rather, we wondered whether gender parity could ever be achieved with current practices. To assess that possibility, we used transition matrices to model faculty flux, and examined convergence behavior to characterize the end state. By assuming the flux fit a semi-Markov process (i.e. that the transition matrix elements are constant), we could calculate the proportions of faculty in each of the three ranks at convergence, when no further change would occur.

**Fig 4:**
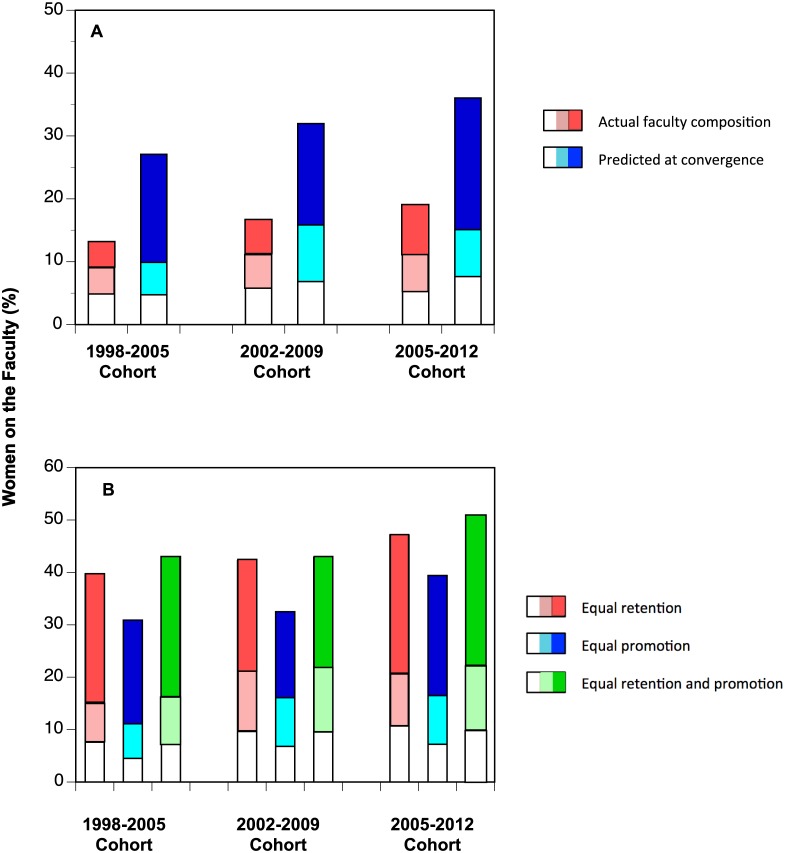
Results of simulations. For all bars, the bottom (white) segment represents Assistant Professors, with Associate Professors above them and Professors as the top segment. A) Actual representation of women on the faculty (red) and convergent state (blue), given the respective transition matrix. B) Simulated convergence states under the assumption that recruitment of Assistant Professors is 50% women (red); 2) retention of male and female Assistant Professors is equal (blue); or 3) both recruitment and retention of Assistant Professors is equal (green).

Fig 4A shows projections for each of the three cohorts. Within each cohort, the red bar represents the initial representation of women on the faculty and the blue represents the convergent state $\tilde{\Omega }$. The simulated end states all show that, given current patterns of recruitment and retention, the proportion of women on the faculty will increase. Furthermore, in each successive cohort we see enhanced representation of women. Finally, representation of women among Professors increases dramatically at convergence for each cohort. Even so, the proportion of women on the faculty does not reach gender parity for any; indeed, women achieve at best a 37% representation at convergence. With current patterns of recruitment and retention, women will *always* be under-represented on the faculty. Demographic inertia may maintain gender gaps in the short run, but we cannot ascribe long-term under-representation of women to that effect alone.

We examined the sensitivity of these projections to gender gaps in faculty flux (Fig 4B). We first artificially inflated recruitment of women Assistant Professors to equal that of men, then equated retention, and then both. The simulated convergence states (Fig 4B) are uniformly closer to gender parity than the current state or anticipated convergent state (cf. Fig 4A).

Equating recruitment of male and female Assistant Professors (red bars in Fig 4B) increases women’s representation at convergence, but never parity. The simulation in which retention gender gaps were erased while maintaining recruitment gaps (blue bars in Fig 4B), again improved women’s representation at convergence, but more weakly. Not surprisingly, bringing both recruitment and retention of Assistant Professors to gender parity produced the best representation of women at convergence (green bars in Fig 4b). Even so, gender parity was reached only for one such simulation. For the third cohort, equating flux of Assistant Professors, coupled with that cohort’s equal rates of promotion to Professor (Fig 2D) produced a future faculty with half women. This simulation illustrates that post-tenure career progression is essential for gender parity as well.

Indeed, comparisons of these simulations across cohorts is particularly instructive to disentangle effects of recruitment and retention across ranks. Recruitment of women to Assistant Professorships was roughly constant across this time frame (Fig 2B). The second cohort showed enhanced recruitment of women to both Associate Professor and Professor ranks, while the third showed vigorous recruitment to the highest rank (Fig 2B). However, higher voluntary resignations of women faculty consistently undercut those recruitment efforts (Fig 2C). Improvement of promotion rates for women Associate Professors (Fig 2D) over time also worked to increase the seniority of women faculty. The end result was our prediction that women would never achieve parity with existing demographic flux (Fig 4A). In only one case does the model predict women’s representation equaling that of men, when rates of recruitment, retention, and promotion are all equal (Fig 4B).

## Conclusions

Demographic inertia predicts that despite vigorous hiring, many years will be needed to allow for retiring men to be replaced by women [4, 9, 28]. The assumption of inertia has placed a spotlight on recruitment of women into junior positions [16]. Demographic inertia may partially explain women’s under-representation in the short term, but over the long term achieving the goal of gender parity will require substantial changes also to existing patterns of retention, and career progression. This particular institution has put many policies in place to address root causes of disparity, and enriching the applicant pool is a key strategy to achieving a faculty with equal representation of women. Yet retention and post-tenure promotion to Professor remain problematic; changing those patterns will require structural and/or cultural change to provide environments that allow everyone to succeed. Achieving gender parity among STEM faculty, which requires equalizing career progression for men and women across their entire careers, depends on addressing all the environmental factors that impede career progression.

## Supporting Information

S1 Text Additional methodology details (DOCX)
