# Supplementary material for: Gender in Science and Engineering Faculties: Demographic Inertia Revisited
Source: PLoS One. 2015 Oct 21;10(10):e0139767. doi: 10.1371/journal.pone.0139767 (PMC4619263; doi:10.1371/journal.pone.0139767)
Supplement: S1 Text — (DOCX) [file pone.0139767.s001.docx]

**S1 Text. Additional methodology details.**

Across five time points, we identified three seven-year-long cohorts: 1998-2005, 2002-2009, and 2005-2012. The seven-year interval was chosen to reflect the expectation that Assistant Professors would have been tenured and promoted, and many Associate Professors would have been promoted to Professor. Our data consisted of demographic snapshots at each time point and personnel changes between time points. We could therefore track the fate of every individual who was present at time *t* and as well as back-track the history of individuals present at time *t+7.* Comparisons of these lists allowed us to separate faculty members into two separate groups: those initially present at the beginning of the seven-year period (time *t*), and those who were subsequently hired (in years *t+1* through *t+6)*.

The snapshots used faculty categories of Assistant, Associate, and full Professors, separated by gender. We first examined individuals present at time *t* and assigned each to a category describing their status 7 years later. For Assistant Professors, these included: Promoted to Associate; Promoted to Full; Denied Tenure; Resigned; Retired/Died; and Still in Rank. Similar transitions were defined for Associate Professors and Professors; note that many transitions are impossible personnel actions (e.g. from Professor to Assistant Professor, from retirement to Associate Professor).

Next, we examined the faculty snapshots at time *t+7* to identify individuals who had not been on the faculty at time *t*, namely those who were recruited during the interval. Again, we kept separate categories for Assistant, Associate, and full Professors, and separated the data by gender.

**Analysis**

*Faculty demographics*

Numbers of faculty by rank and gender across the five time points are given in the main paper. We then examined the flux of faculty through the 6 possible states identified in Table S1 as well as influx of new hires, separately for each cohort. We used a model similar to classical Markov Chain models on finite states in order to estimate representation of female and male Assistant, Associate, and full Professors after many iterations.

We let $\omega_{1}(t)$ be the number of Assistant Professors, $\omega_{2}(t)$ be the number of Associate Professors, $\omega_{3}(t)$ be the number of full Professors, $\omega_{4}(t)$ be the number who will be denied tenure, $\omega_{5}(t)$ be the number who will resign, and $\omega_{6}(t)$ be the number who will retire (or die). Then after 7 years, these variables, satisfy the following matrix equation

$$\Omega(t+7)=A\Omega(t)+B$$

where $\Omega(t)$ is the column vector of the $\omega_{i}(t)$ values. The column vector $B$ represents new individuals entering the faculty and has elements $\upsilon_{1}$ (Assistant Professors to be hired during the interval), $\upsilon_{2}$ (Associate Professors) and $\upsilon_{3}$ (full Professors), with the remaining elements equal to 0 because we do not recruit into the states of tenure denial, resignation, and retirement/death.

In the transition matrix A, columns correspond to initial states and rows to the states at the end of a seven-year time period. Once a Professor is denied tenure, resigns or retires, he or she remains in this state (that is, these are absorbing states). Consequently, for our data, $A$is lower triangular (entries above the diagonal are zero) and the lower right submatrix is the 3x3 identity matrix.

To estimate a convergence state, we must assume that the faculty hiring and promotion behavior remains constant in subsequent time periods. Note that convergence cannot occur if one of the entries along the diagonal is 1. Furthermore, the number of professors who retire/die should tend to infinity as time increases (because these are absorbing states). To avoid that trivial result, we reduce the matrix A to and the vector B to to include only the three faculty ranks. With those modifications and conditions, we can see that

where *I* is the 3x3 identity matrix (our conditions on the diagonal imply that is invertible).

Further, as $t\to\infty$, each entry of tends to zero, so the limiting distribution of the numbers of faculty at each rank tends to the following

Note again that the matrix and vector have tildes to denote that they are of reduced rank, because they do not include the three absorbing states of retirement, resignation, and tenure denial.

We computed the convergence states via the final equation above in Mathematica, which gave the predicted numbers of individuals in each rank by gender; from those numbers we calculated percentages that are reported in the main article.
